# Maternal Capabilities Are Associated with Child Caregiving Behaviors Among Women in Rural Zimbabwe

**DOI:** 10.1093/jn/nxaa255

**Published:** 2020-11-19

**Authors:** Cynthia R Matare, Mduduzi N N Mbuya, Katherine L Dickin, Mark A Constas, Gretel Pelto, Bernard Chasekwa, Jean H Humphrey, Rebecca J Stoltzfus, for the Sanitation Hygiene Infant Nutrition Efficacy (SHINE) Trial Team, Prendergast Andrew

**Affiliations:** Program in International Nutrition, Division of Nutritional Sciences, Cornell University, Ithaca, NY, USA; Zvitambo Institute for Maternal and Child Health Research, Harare, Zimbabwe; Program in International Nutrition, Division of Nutritional Sciences, Cornell University, Ithaca, NY, USA; Zvitambo Institute for Maternal and Child Health Research, Harare, Zimbabwe; Global Alliance for Improved Nutrition, Washington, DC, USA; Program in International Nutrition, Division of Nutritional Sciences, Cornell University, Ithaca, NY, USA; Charles H. Dyson School of Applied Economics and Management, Cornell University, Ithaca, NY, USA; Program in International Nutrition, Division of Nutritional Sciences, Cornell University, Ithaca, NY, USA; Zvitambo Institute for Maternal and Child Health Research, Harare, Zimbabwe; Zvitambo Institute for Maternal and Child Health Research, Harare, Zimbabwe; Department of International Health, Johns Hopkins Bloomberg School of Public Health, Baltimore, MD USA; Program in International Nutrition, Division of Nutritional Sciences, Cornell University, Ithaca, NY, USA

**Keywords:** maternal capabilities, caregiving, depression, gender norms attitudes, Zimbabwe

## Abstract

**Background:**

Young children require high-quality care for healthy growth and development. We defined “maternal capabilities” as factors that influence mothers’ caregiving ability (physical and mental health, social support, time, decision-making autonomy, gender norm attitudes, and mothering self-efficacy), and developed survey tools to assess them.

**Objectives:**

We hypothesized that mothers with stronger capabilities during pregnancy would be more likely to practice improved care behaviors after their child was born.

**Methods:**

We assessed maternal capabilities among 4667 pregnant women newly enrolled in the Sanitation Hygiene Infant Nutrition Efficacy (SHINE) trial. Several improved child-care practices were promoted until 18 mo postpartum, the trial endpoint. Care practices were assessed by survey, direct observation, or transcription from health records during postpartum research visits. We used logistic regression to determine the predictive association between maternal capabilities during pregnancy and child-care practices.

**Results:**

Mothers with more egalitarian gender norm attitudes were more likely to have an institutional delivery [adjusted OR (AOR), 2.06; 95% CI, 1.57–2.69], initiate breastfeeding within 1 h of delivery (AOR, 1.38; 95% CI, 1.03–1.84), exclusively breastfeed (EBF) from birth to 3 mo (AOR, 2.55; 95% CI, 1.95–3.35) and 3–6 mo (AOR, 1.75; 95% CI, 1.36–2.25), and, among households randomized to receive extra modules on sanitation and hygiene, have soap and water at a handwashing station (AOR, 1.76; 95% CI, 1.29–2.39). Mothers experiencing time stress were less likely to EBF from birth to 3 mo (AOR, 0.79; 95% CI, 0.66–0.93). Greater social support was associated with institutional delivery (AOR, 1.53; 95% CI, 1.37–1.98) and, among mothers randomized to receive extra complementary feeding modules, feeding children a minimally diverse diet (AOR, 1.18; 95% CI, 1.01–1.37). Depressed mothers were 37% and 33%, respectively, less likely to have an institutional delivery (AOR, 0.63; 95% CI, 0.44–0.88) and a fully immunized child (AOR, 0.67; 95% CI, 0.50–0.90).

**Conclusions:**

Interventions to reduce maternal depression, time stress, inadequate social support, and inequitable gender norms may improve maternal child caregiving.

See corresponding commentary on page 455.

## Introduction

Child undernutrition remains a major public health challenge globally, with 21.9%, 7.3%, and 41.7% of children under 5 years old stunted, wasted, and anemic, respectively (1, 2). Despite making substantial strides in the past 2 decades, the projected rate of improvement will be too slow to “end all forms of malnutrition” (Sustainable Development Goal 2) or achieve a “50% reduction in the number of children under 5 who are stunted” (the World Health Assembly global target) by 2030 (3, 4). Moreover, implementing only the current evidence-based strategies is unlikely to achieve these goals. The 2013 Maternal and Child Nutrition Study Group (5) estimated that scaling up 10 recommended nutrition-specific interventions to 90% coverage in the highest-burden countries would, rather modestly, reduce stunting in children under 5 by 20%. The same group also identified “nutrition-sensitive” interventions to address underlying determinants of undernutrition (e.g., agriculture, social safety net, early child development, and parental education), but concluded that evidence of the nutritional benefits of such investments is scarce or conflicting (5). Thus, new intervention targets are needed.

Infants and young children are completely dependent on others, primarily their mothers, for nutrition, safe housing, and preventive and curative health care; indeed, the UNICEF framework on malnutrition highlights “nurturing care” as a critical requirement for healthy child growth and development (6). To provide good child care, a mother must have access to resources (money, food, health care, knowledge, and skills, some of which are acquired through education). However, resources alone are not enough: money must be prioritized for child needs, available food must be appropriately prepared and fed to young children, and knowledge and skills must be practiced. We sought to identify and assess the underlying characteristics of a mother that determine her ability to provide high-quality child care.

## Methods

### Maternal Capability Survey Tool

Building on the UNICEF model of care (7), the human capabilities theory (8), and empirical studies, we developed a maternal capabilities construct that has been described in detail (9). Briefly, we began by identifying 7 characteristics of women that have been shown to be associated with women's productivity, quality of life, scope of responsibility and authority, and/or with child-care behaviors in previous studies. We defined these characteristics as “maternal capabilities,” and provide brief justifications:

1. Decision-making autonomy: maternal decision-making autonomy refers to the control or influence a mother has over choices that affect her family and herself. Examples include authority on how family income is spent and independence in going outside the household unaccompanied and without other's approval (10). A systematic review of 22 studies concluded that the association between maternal autonomy and child nutritional status is generally positive, but varies by how autonomy is defined and assessed; in particular, mothers’ control, specifically over family health-care decisions, has been most consistently associated with children's nutritional status (11). In Chad, maternal autonomy in making child-feeding decisions was significantly associated with child height-for-age *z*-scores (12).
2. Gender norm attitudes: maternal belief that men and women should have equal access to resources and opportunities (i.e., egalitarian gender norm attitudes) is emerging as an important determinant of good child care. Mothers with greater egalitarian gender norm attitudes are more likely to give birth in a health institution (13), have fully immunized children (13), and practice exclusive breastfeeding (EBF) during early infancy (14).
3. Mental health: poor maternal mental health, usually measured as depression, is associated with symptoms (loss of interest or pleasure in usual activities, decreased energy, feelings of guilt or low self-worth, disturbed sleep or appetite, and poor concentration) that can have a negative impact on child caregiving behaviors (15). Depression reduces positive maternal-child interaction (16) and child health care–seeking behavior (17). In a systematic review, maternal depression was associated with early childhood underweight and stunting (18).
4. Mothering self-efficacy: mothering self-efficacy reflects a woman's self confidence in her role as a competent mother (19). Self efficacy is included in many health behavior models, often as the proximal determinant of behavior (20–22). There is particularly strong evidence for the central role of mothering self-efficacy in optimal breastfeeding behaviors (23, 24).
5. Physical health: women in low- and middle-income countries (LMICs) face a high burden of infectious disease, nutritional deficiencies, and obstetric and gynecologic disorders (25). Illness may reduce mothers’ energy levels, thereby affecting the quality of child care they provide (7).
6. Social support: social support is included in many models of health behavior, including Bronfenbrenner's (26) ecological systems theory. Many studies have demonstrated the importance of social support, particularly for breastfeeding (27), and the child health benefits of emotional and informational social support provided by peer counselors (28).
7. Time stress: women in LMICs work long hours and have little leisure time (29); these women's time has been described as a 0-sum game: new activities can only be added at the expense of others (7). Consequently, workload can negatively influence how well mothers care for their children, especially when child care is less prioritized compared to other household tasks.

Second, we designed a quantitative survey to assess the capabilities. Questions to assess “time stress” were newly developed; survey items from previously published survey instruments were adapted to assess decision-making autonomy (30), gender norm attitudes (31), mothering self-efficacy (32, 33), perceived physical health (34), mental health (35), and social support (36, 37). Adaptation included modifying survey items to make them contextually relevant for pregnant women in a LMIC setting, or dropping survey items that could not be modified (9). We defined strong (positive) maternal capabilities as good physical and mental health, high levels of social support and mothering self-efficacy, high autonomy for decision making within the household, egalitarian gender norms, and low levels of perceived time stress (9).

The Maternal Capabilities Tool (available at https://osf.io/w93hy; **Table 1**) was administered within the Sanitation Hygiene Infant Nutrition Efficacy (SHINE) trial in rural Zimbabwe. Prior to the trial, the Maternal Capabilities Tool was translated into 2 local languages and pretested using cognitive interviewing techniques among women in rural Zimbabwe.

**TABLE 1:** Metric characteristics, source, and example questions of maternal capabilities domains

Domain	Cronbach's alpha	Possible scores	Source	Example question	Interpretation of high score
Decision-making autonomy: 5 questions (responses coded 0 = no, 1 = yes); score calculated as sum of 5 responses.	0.83	0–5	Questions adapted from the Zimbabwe Demographic Health Survey (30)	Can you decide on your own to buy medicine for yourself?	Greater decision-making autonomy
Gender norm attitudes: 6 questions (responses coded on 5-point Likert Scale where 1 = restrictive gender norm attitudes and 5 = egalitarian gender norm attitudes); score calculated as mean of 6 responses.	0.87	1–5	Questions adapted from the Gender Norm Attitudes Scale (31)	A woman must accept that her husband or partner beats her, in order to keep the family together.	More egalitarian gender norm attitudes
Maternal depressive symptoms: 10 questions (various responses coded on 4-point Likert Scale where 0 = lowest levels of depressive symptoms and 3 = highest levels of depressive symptoms); score calculated as sum of 10 responses.	0.82	0–30	All 10 questions of Edinburgh Postnatal Depression Scale used (35)	Have you been so unhappy that you have had difficulty sleeping?	High levels of depressive symptoms
Mothering self-efficacy: 6 questions (responses coded on 5-point agreement Likert Scale where 1 = low self-efficacy and 5 = high self-efficacy); score calculated as mean of 6 responses.	0.63	1–5	Questions adapted from the Parenting Sense of Competence Scale (32) and the Parenting Self-Agency Measure (33)	I know how to do all that is required to be a good mother to my child.	Greater mothering self-efficacy
Perceived health status: 11 questions (various response codes used for different questions). Responses recoded on a 0–5 scale with 0 = least healthy and 5 = most healthy. Score calculated as per source instructions where final score is calculated as mean of subscores.	0.81	0–5	Adapted from the RAND 36-Item Health Survey (34)	I seem to get sick more often than other people.	Perceives having better physical health
Perceived social support: 16 questions (responses coded on 5-point frequency Likert Scale, where 1 = low levels of social support, 5 = high levels of social support); score calculated as mean of 16 responses.	0.78	1–5	Questions adapted from the Interpersonal Support Evaluation List (36) and the Medical Outcomes Study Social Support Survey (37)	If you needed money in an emergency, such as the need to take a sick person to the hospital, how often could you count on someone's help?	Perceives having more social support
Perceived time stress: 5 questions (5-point Likert Scale where 1 = low levels of stress and 5 = high levels of stress); score calculated as mean of 5 responses.	0.77	1–5	Questions developed for SHINE trial	I worry about all the things that I have to get done in a day.	Perceives having high levels of time stress

Abbreviation: SHINE, Sanitation Hygiene Infant Nutrition Efficacy.

### SHINE trial

Methods and primary outcomes of the SHINE trial have been previously reported (38–40). Briefly, SHINE was a cluster-randomized, community-based trial carried out in 2 contiguous rural districts in Zimbabwe (Chirumanzu and Shurugwi) in Midlands province. Clusters, defined as a catchment area of between 1 and 4 village health workers (VHWs) employed by the Zimbabwe Ministry of Health and Child Care, were randomly allocated to 1 of 4 treatment groups: Standard Of Care; water, sanitation, and hygiene (WASH); infant and young child feeding (IYCF); or WASH + IYCF. Women were enrolled during pregnancy between 22 November 2012 to 27 March 2015 at a median gestational age of 12.5 wk (IQR, 9–16), following written informed consent. Interventions promoting specific child-care practices were delivered by VHWs during monthly home visits. Research nurses made home visits at baseline (∼2 wk after enrolment), 32 weeks’ gestation, and infant ages 1, 3, 6, 12, and 18 mo. At the 18-mo visit, mothers and infants were visited anywhere in Zimbabwe. Intermediate visits were conducted only when the mother was available in the household where she consented, because the interventions were household-based.

At baseline, a structured questionnaire was used to elicit maternal age, years of completed schooling, marital status, parity, child dependency ratio (number of children under the age of 18 y for whom the participant was the primary caregiver), and household size. We also determined participants’ level of satisfaction with the work of their VHW. Household food security status was measured using a 12-item Coping Strategies Index, adapted from Maxwell (41). A measure of relative household wealth was constructed as described (42). Finally, maternal capabilities were assessed at the baseline visit using the newly developed Maternal Capabilities Survey Tool. Mothers with an Edinburgh Postnatal Depression Scale (EPDS) score ≥12 and/or suicidal ideation were defined as clinically depressed, a definition previously validated by psychometric testing among Zimbabwean women (43); these women were referred to local clinics for further assessment. Women were tested for HIV; those testing positive were urged to seek immediate antenatal care for prevention of mother-to-child transmission.

During postnatal home visits, child-care behaviors that were promoted in 1 or more arms of the SHINE trial were assessed. Institutional delivery, breastfeeding, and timely immunization were promoted across all 4 arms of the SHINE trial. Place of delivery and child immunization status were transcribed from the child's health records, or confirmed by the mother if the health records were not available. Early initiation of breastfeeding was assessed at the 1-mo visit through a single question asking the mother how soon after delivery she put her child to the breast. Mothers’ responses of <1 h were classified as early initiation. EBF from birth up to 3 months of age (early EBF) was assessed at the 1-mo and 3-mo visits, and EBF from 3 months up to 6 months of age (late EBF) was assessed at the 3-mo and 6-mo visits using a tool that was developed in Zimbabwe and has been described previously (44, 45). Infants were classified as EBF if they received only breastmilk in the previous 24 h. Dietary diversity was assessed for infants in the IYCF arms at the 12-mo visit using a dietary questionnaire; children were classified as having a minimally diverse diet if they consumed food from at least 4 food groups in the previous 24 h (46). Handwashing was promoted in the WASH arms; handwashing practice was assessed by observation for the presence of soap and water at the handwashing station at the 12-mo visit.

A priori, we hypothesized that mothers with stronger maternal capabilities during pregnancy would be more likely to practice the child-care behaviors promoted in the SHINE trial after their child was born (47, 48). Additionally, we hypothesized that the monthly home visits by VHWs would have positive effects on some caregiver capabilities, particularly time stress, mental health, social support, and mothering self-efficacy. In Haiti, regular visits by community-level workers improved mothers’ self-rated physical and mental health and time stress (49).

Finally, as 1 indicator of the performance of the maternal capabilities tool, we investigated whether the individual survey tools measured discrete aspects of women's child-care capability. First, we examined the strength and significance of correlation coefficients between capabilities; second, we compared independent socioeconomic and demographic determinants of each capability.

### Statistical analysis

All analyses were performed using STATA version 14 (StataCorp LP, College Station, TX). The EPDS scores were modeled as depressed (score ≥12 and/or suicidal ideation) or not depressed (43). Decision-making autonomy was calculated as the sum of 5 questions, each of which could be scored as 0 (no) or 1 (yes). The median decision-making autonomy score was 5 (IQR, 4–5) out of a range of possible scores of 0–5; accordingly, we modeled this variable as a binary variable, where 1 indicated a score of 5, and 0 indicated a score <5. For each of the other maternal capabilities, the mean of several Likert-type items was calculated to give a composite score.

A correlational matrix was constructed to assess the strength of association of every pair of maternal capabilities using nonparametric (Spearman's *ρ*) correlation. Stepwise multivariable regression models were constructed to identify associated demographic and socioeconomic determinants of each capability. The presence of maternal depression and high compared to lower decision-making autonomy were analyzed using logistic regression, and all other capabilities were analyzed using linear regression.

All child-care behavior variables were binary. We used logistic regression with cluster-robust inference, adjusted for SHINE trial arm to identify the unadjusted association of each capability with each child-care practice. Models predicting child diet diversity were limited to women in the IYCF arms, and models predicting handwashing were limited to women in the WASH arms. In adjusted analyses, we controlled for prespecified baseline covariates (maternal age, education, and religion; household wealth; and maternal relationship with her VHW), after determining that the variable was associated with both the care behavior and the capability in a univariable regression, with statistical significance at *P* < 0.2.

### Ethical approval

This study was approved by the Medical Research Council of Zimbabwe and the Johns Hopkins University Bloomberg School of Public Health Institutional Review Board. Written informed consent was obtained from all participants, in a language of their choice (English, Ndebele, or Shona), prior to the baseline interview.

## Results

A total of 4667 women provided maternal capability information at the baseline visit (∼14 weeks’ gestation; **Supplemental Figure 1**). Of these, 2347, 2617, and 3181 mother-infant pairs were available for follow-up in their homesteads at the 1-mo, 3-mo, and 12-mo visits, respectively, and provided information on early initiation and exclusivity of breastfeeding. Infant dietary diversity (IYCF arms) and the presence of soap and water (WASH arms) were assessed at the 12-m visit. Follow-up was low at the 1- and 3-mo visits, primarily because of the cultural practice (*kusungira*) for women (especially primiparous) to return to their parental home during the perinatal period (14, 50). At the 18-mo visit, when children were visited anywhere in the country to obtain primary outcome data, 4058 provided immunization information.

Women had a mean age of 26.3 (SD, 6.7) y and relatively high levels of education (mean, 9.5; SD, 1.8 y), which is typical of Zimbabwe (**Table 2**) (30). Only 9 participants (<1%) had never been to school, while 43% had completed 11 y of formal schooling. The HIV prevalence was 16.5%.

**TABLE 2:** Sociodemographic characteristics and maternal capabilities of pregnant women living in rural Zimbabwe and their child caregiving behaviors between birth and 18 months’ postpartum

Sociodemographic characteristics	Value
Age, y	26.3 (6.7)
15–18.9, *n*/*N* (%)	688/4472 (15.4)
19–24.9, *n*/*N* (%)	1451/4472 (32.5)
25–34.9, *n*/*N* (%)	1788/4472 (40.0)
≥35, *n*/*N* (%)	545/4472 (12.2)
Education, y	9.5 (2.5)
≤7, *n*/*N* (%)	865/4482 (193.0)
8–10, *n*/*N* (%)	1655/4482 (36.9)
≥11, *n*/*N* (%)	1962/4482 (43.8)
Married, *n*/*N* (%)	4247/4444 (95.6)
Parity, *n*/*N* (%)	2 [1–3]
Employed, *n*/*N* (%)	397/4667 (8.5)
Child dependency ratio	1.5 (1.5)
Household occupants	4 [3–6]
HIV infected, *n*/*N* (%)	773/4676 (16.5)
Maternal capabilities^1^, ^2^	
Decision-making autonomy, score, range: 0–5	5 [4–5]
Takes part in 5/5 decisions, *n*/*N* (%)	2356/4232 (55.7)
Depressed, EPDS ≥12 and/or suicidal, score, range: 0–30, *n*/*N* (%)	395/4574 (8.6)
Gender norm attitudes, score, range: 1–5	3.1 (0.5)
Mothering self-efficacy, score, range: 1–5	4.0 (0.4)
Perceived health status, score, range: 0–5	3.5 (1.0)
Perceived social support, score, range:1–5	3.6 (0.6)
Perceived time stress, score, range: 1–5	2.7 (0.7)
Child caregiving behaviors	
Had an institutional delivery, *n*/*N* (%)	3418/3799 (90.0)
Initiated breastfeeding within 1 h of delivery, *n*/*N* (%)	1938/2232 (86.8)
Exclusively breastfed birth to 3 mo of age, *n*/*N* (%)	1916/2348 (81.6)
Exclusively breastfed 3 to 6 mo of age, *n*/*N* (%)	2169/2621 (82.8)
Child fully immunized by 18 mo of age, *n*/*N* (%)	3561/4058 (87.8)
Child's diet minimally diverse at 12 months of age,^3^ *n*/*N* (%)	1088/1558 (69.8)
Water and soap present at handwashing station at 12 mo postpartum,^4^ *n*/*N*, (%)	1251/1494 (83.7)

Values are mean (SD) or median [IQR], unless otherwise indicated. Abbreviations: EPDS, Edinburgh Postnatal Depression Scale; SHINE, Sanitation Hygiene Infant Nutrition Efficacy.

1 Stronger maternal capabilities are not having depression and having low time stress; for all other capabilities, higher values represent stronger capability (more egalitarian gender norm attitudes; greater mothering self-efficacy; better physical health; and more social support).

2 Possible denotes the possible range of scores for each test.

3 Infant and young child feeding arms of the SHINE trial only.

4 Water, sanitation, and hygiene arms of the SHINE trial only.

Mean (SD) or median (IQR) scores for each maternal capability are shown in Table 2. A total of 395 (8.6%) participants were defined as clinically depressed and referred for further assessment. As previously noted, 75% of women had a score of 4 or 5 in decision-making autonomy, indicating that most women perceived they were empowered to make most household decisions. On average, participants had relatively high levels of mothering self-efficacy and scores near the midpoints of the possible ranges for gender norm attitudes, perceptions of their health, social support, and time stress.

### Associations between maternal capabilities

The correlation coefficients between pairs of capabilities were mostly statistically significant, but relatively weak (all correlation coefficients, ≤0.30; **Table 3**). The strongest correlations were between maternal depression and physical health status (−0.26), social support (−0.25), and time stress (0.30); and between time stress and physical health status (−0.27) and social support (−0.23).

**TABLE 3:** Spearman's Rank correlations of maternal capabilities with each other among rural Zimbabwean pregnant women

	Decision-Making Autonomy	Gender Norm Attitudes	Maternal Depressive Symptoms	Mothering Self-Efficacy	Perceived Health Status	Perceived Social Support	Perceived Time Stress
Decision-Making Autonomy	1.00 (4232)	—	—	—	—	—	—
Gender Norm Attitudes	0.17*** (4197)	1.00 (4619)	—	—	—	—	—
Maternal Depressive Symptoms	−0.11*** (4149)	−0.16*** (4530)	1.00 (4573)	—	—	—	—
Mothering Self-Efficacy	0.12*** (4167)	0.05** (4552)	−0.04* (4508)	1.00 (4598)	—	—	—
Perceived Health Status	0.05** (4167)	0.00 (4543)	−0.26*** (4497)	0.04* (4524)	1.00 (4589)	—	—
Perceived Social Support	0.15*** (4086)	0.16*** (4455)	−0.25*** (4413)	0.16*** (4435)	0.16*** (4426)	1.00 (4498)	—
Perceived Time Stress	−0.04** (4119)	−0.14*** (4497)	0.30*** (4455)	0.02 (4477)	−0.27*** (4468)	−0.23*** (4381)	1.00 (4542)

Data are shown as correlations (*n*). Stronger maternal capabilities are not having depression and having low time stress; for all other capabilities, higher values represent stronger capability (more egalitarian gender norm attitudes; greater mothering self-efficacy; better physical health; and more social support). *P < 0.05,  **P < 0.01,  ***P < 0.001.

### Sociodemographic determinants of maternal capabilities

In fully adjusted models, food security was associated with stronger capabilities in all 7 domains, and maternal education was positively associated with stronger capabilities in 5 of the 7 domains. Mothers who reported being satisfied with the performance of their VHW had higher mothering self-efficacy and social support scores, and lower time stress scores. Other socioeconomic determinants were more variable between capabilities (**Table 4**). For example, older age was associated with stronger capabilities for 3 domains (decision-making autonomy, gender norm attitudes, and mothering self-efficacy), but weaker capability for time stress (i.e., older mothers reported higher levels of time stress). A higher child dependency ratio was associated with greater decision-making autonomy and greater mothering self-efficacy, but poorer perceived physical health and greater time stress. Household wealth was positively associated with greater decision-making autonomy, self-efficacy, and social support, but gender norm attitudes, perceived health status, time stress, and depression were all independent of wealth. Having a satisfactory relationship with the VHW who visited was associated with greater mothering self-efficacy, greater social support, and lower time stress.

**TABLE 4:** Association between sociodemographic factors and maternal capabilities in rural Zimbabwean women determined by stepwise multivariable regression

	High decision-making autonomy	Depression	Gender Norm Attitudes	Mothering Self-Efficacy	Perceived Health Status	Perceived Social Support	Perceived Time Stress
	*n* = 3415	*n* = 3682	*n* = 3665	*n* = 3619	*n* = 3709	*n* = 3543	*n* = 3566
	OR (95% CI)	OR (95% CI)	β ± SE	β ± SE	β ± SE	β ± SE	β ± SE
Household size	0.95 (0.90–0.98)**	1.07 (1.02– 1.13)*	…	−0.01 ± 0.00*	…	…	…
Child dependency ratio	1.15 (1.08–1.23)***	…	…	0.02 ± 0.01***	−0.04 ± 0.01**	…	0.04 ± 0.01***
Maternal age, y							
15–18.9	0.60 (0.47–0.79)***	…	−0.20 ± 0.03***	−0.22 ± 0.02***	…	…	−0.10 ± 0.04*
19–24.9	0.77 (0.64–0.95)*	…	−0.09 ± 0.02***	−0.08 ± 0.02***	…	…	−0.09 ± 0.03**
25–34.9	Ref.	…	Ref.	Ref.	…	…	Ref.
≥35	1.49 (1.16–1.92)**	…	0.02 ± 0.03	0.02 ± 0.02	…	…	0.12 ± 0.04**
Education, y							
≤7	0.62 (0.51–0.75)***	1.49 (1.09–2.04)*	−0.19 ± 0.02***	…	0.08 ± 0.05	−0.11 ± 0.03***	0.12 ± 0.04**
8–10	0.78 (0.65–0.94)**	1.42 (1.10–1.86)**	−0.08 ± 0.02***	…	0.06 ± 0.04	−0.06 ± 0.02**	0.05 ± 0.03
≥11	Ref.	Ref.	Ref.	…	Ref.	Ref.	Ref.
Marital status							
No	Ref.	Ref.	Ref.	Ref.	Ref.	Ref.	Ref.
Yes	0.74 (0.53–1.01)	0.58 (0.38–0.89)*	−0.14 ± 0.05**	0.18 ± 0.04***	−0.15 ± 0.08	−0.07 ± 0.04	0.16 ± 0.05**
Wealth status							
Lowest	Ref.	…	…	Ref.	…	Ref.	…
Lower	1.17 (0.92–1.48)	…	…	0.03 ± 0.02	…	0.06 ± 0.03*	…
Middle	1.28 (0.99–1.67)	…	…	0.06 ± 0.02**	…	0.01 ± 0.03	…
Higher	1.11 (0.86–1.44)	…	…	0.06 ± 0.02**	…	0.07 ± 0.03*	…
Highest	1.41 (1.07–1.87)*	…	…	0.07 ± 0.02**	…	0.09 ± 0.03**	…
Food security status							
Low	Ref.	Ref.	Ref.	Ref.	Ref.	Ref.	Ref.
Middle	1.09 (0.89–1.34)	0.56 (0.40–0.78)***	0.11 ± 0.03***	0.02 ± 0.02	0.15 ± 0.05**	0.09 ± 0.03**	−0.19 ± 0.04***
High	1.32 (1.12–1.56)**	0.36 (0.27–0.48)***	0.18 ± 0.02***	0.05 ± 0.02**	0.34 ± 0.04***	0.22 ± 0.02***	−0.29 ± 0.03***
HIV status							
Negative	…	Ref.	…	…	…	Ref.	Ref.
Positive	…	1.72 (1.23–2.33)***	…	…	…	−0.07 ± 0.02**	0.08 ± 0.04*
Satisfied with village health worker	…	…	…	0.24 ± 0.02***	…	0.17 ± 0.02***	−0.06 ± 0.02**
Parity	…	1.49 (1.21–1.73)*	−0.05 ± 0.02*	−0.05 ± 0.02*	…	−0.06 ± 0.03*	…

Logistic regression was used for bivariate outcomes (decision-making autonomy and depression); linear regression was used for other outcomes. Forward stepwise procedures were used. Stronger maternal capabilities are not having depression and having low time stress; for all other capabilities, higher values represent stronger capability (more egalitarian gender norm attitudes; greater mothering self-efficacy; better physical health; and more social support). Cells with ellipses signify that the variable was not added to the model during the forward selection procedure. *P < 0.05,  **P < 0.01,  ***P < 0.001.

### Associations between maternal capabilities and child caregiving behaviors

Maternal decision-making autonomy during pregnancy was not associated with any of the care behaviors investigated. Mothers who were clinically depressed were 37% less likely to have an institutional delivery [adjusted OR (AOR), 0.63; 95% CI, 0.44–0.88] and 33% less likely to have their child fully immunized (AOR, 0.67; 95% CI, 0.50–0.90). Mothers with more egalitarian gender norm attitudes were more likely to have an institutional delivery (AOR, 2.06; 95% CI, 1.57–2.69), initiate breastfeeding early (AOR, 1.44; 95% CI, 1.01–2.06), and practice early (AOR, 2.55; 95% CI, 1.95–3.35) and late (AOR, 1.75; 95% CI, 1.36–2.25) exclusive breastfeeding (**Table 5**). There was weaker evidence that women with more egalitarian gender norm attitudes were also more likely to have a fully immunized child (AOR, 1.20; 95% CI, 0.98–1.46). There was weak evidence that mothers were more likely to exclusively breastfeed during the 1–3-mo interval if they perceived themselves to be in better physical health (AOR, 1.13; 95% CI, 1.00–1.29). Greater mothering self-efficacy was weakly associated with early breastfeeding initiation (AOR, 1.48; 95% CI, 0.99–2.22) and exclusively breastfeeding during the 3–6-mo interval (AOR, 1.31; 95% CI, 0.98–1.74). Finally, mothers reporting higher levels of time stress were 21% less likely to practice early exclusive breastfeeding (AOR, 0.79; 95% CI, 0.66–0.93). Among women in the IYCF arms of the SHINE trial, those with greater social support were more likely to feed their child a minimally diverse diet (AOR, 1.18; 95% CI: 1.01–1.37). Among women in the WASH arms of the SHINE trial, those having more egalitarian gender norm attitudes were more likely to have water and soap at the handwashing station (AOR, 1.76; 95% CI, 1.29–2.39).

**TABLE 5:** Associations between maternal capabilities during pregnancy and subsequent child caregiving behaviors among rural Zimbabwean women

	Child caregiving behavior						
	Institutional Delivery	Early breastfeeding initiation	Early exclusive breastfeeding	Late exclusive breastfeeding	Child fully immunized	Infant fed diverse diet^1^	Soap and water at handwashing station^2^
Decision-making autonomy							
Unadjusted OR (95% CI); *P* value	1.36 (1.07–1.73); 0.013	1.22 (0.90–1.65); 0.196	1.07 (0.81–1.40); 0.641	1.03 (0.79–1.33); 0.850	1.33 (1.09–1.62); 0.005	1.13 (0.96–1.33); 0.135	1.23 (0.92–1.63); 0.157
Adjusted OR (95% CI); *P* value	1.28 (0.99–1.65); 0.057	—	—	—	1.13 (0.91–1.31); 0.270	—	—
Maternal depressive symptoms							
Unadjusted OR (95% CI); *P* value	0.58 (0.42–0.80); 0.001	0.83 (0.50–1.38); 0.474	0.70 (0.45–1.07); 0.098	0.76 (0.50–1.16); 0.207	0.69 (0.52–0.93); 0.014	0.85 (0.64–1.19); 0.249	1.10 (0.66–1.82); 0.697
Adjusted OR (95% CI); *P* value	0.63 (0.44; 0.88); 0.008	—	—	—	0.67 (0.50–0.90); 0.008	—	—
Gender norm attitudes							
Unadjusted OR (95% CI); *P* value	2.14 (1.65–2.79); 0.000	1.39 (1.04, 1.84); 0.024	2.55 (1.95–3.35); 0.000	1.76 (1.36–2.28); 0.000	1.41 (1.17–1.70); 0.000	1.03 (0.89–1.19); 0.673	1.68 (1.25–2.26); 0.001
Adjusted OR (95% CI); *P* value	2.06 (1.57–2.69); 0.000	1.38 (1.03–1.85); 0.032	2.55 (1.95–3.35); 0.000	1.75 (1.36–2.25); 0.000	1.20 (0.98–1.46); 0.076	—	1.76 (1.29–2.39); 0.000
Mothering self-efficacy							
Unadjusted OR (95% CI); *P* value	1.02 (0.79–1.33); 0.866	1.52 (1.03–2.23); 0.033	1.04 (0.77–1.40); 0.786	1.35 (1.03–1.78); 0.028	1.41 (1.13–1.75); 0.002	1.07 (0.90–1.28); 0.424	1.08 (0.82–1.44); 0.556
Adjusted OR (95% CI); *P* value	—	1.48 (0.99–2.22); 0.054	—	1.31 (0.98–1.74); 0.065	1.13 (0.91–1.41); 0.266	—	—
Health status							
Unadjusted OR (95% CI); *P* value	0.91 (0.81–1.03); 0.139	0.99 (0.86–1.13); 0.838	1.13 (1.00–1.29); 0.055	1.08 (0.97–1.21); 0.171	1.00 (0.92–1.11); 0.85	0.99 (0.91–1.07); 0.856	0.997 (0.89–1.11); 0.965
Adjusted OR (95% CI); *P* value	—	—	—	—	—	—	—
Social support							
Unadjusted OR (95% CI); *P* value	1.65 (1.37–1.98); 0.000	1.08 (0.82–1.42); 0.578	1.27 (0.98–1.63); 0.068	1.07 (0.82–1.39); 0.608	1.21 (1.04–1.41); 0.016	1.23 (1.08–1.42); 0.003	0.88 (0.81–1.28); 0.160
Adjusted OR (95% CI); *P* value	1.53 (1.25–1.86); 0.000	—	—	—	1.10 (0.93–1.29); 0.260	1.18 (1.01–1.37); 0.039	—
Time stress							
Unadjusted OR (95% CI); *P* value	0.81 (0.68–0.96); 0.013	0.98 (0.81–1.19); 0.849	0.79 (0.66–0.93); 0.006	0.90 (0.77–1.06); 0.202	0.96 (0.84–1.10); 0.581	1.03 (0.94–1.13); 0.500	0.94 (0.78–1.13); 0.516
Adjusted OR (95% CI); *P* value	0.84 (0.70–1.00); 0.052	—	0.79 (0.66–0.93); 0.006	—	—	—	—

Higher values represent greater decision-making autonomy; more liberal gender norm attitudes; high levels of depressive symptoms; greater mothering self-efficacy; perceptions of better physical health; perceptions of more social support; and perceptions of high levels of time stress. Data were estimated using univariable logistic regression and controlling for maternal age, maternal education, wealth, religion, and/or relationship with their VHW if these were associated with both the dependent variable and independent variable at *P* < 0.2. For early exclusive breastfeeding, none of the baseline variables considered were associated at *P* < 0.2; therefore, the adjusted models are the same as the unadjusted models. The covariates included in each adjusted model for each maternal capability and each caregiving behavior are listed in parentheses.

For decision-making autonomy, institutional delivery (education, wealth, VHW, religion); breastfeeding initiation (religion); late exclusive breastfeeding (age); child immunized (age, education, wealth, VHW, religion); diverse diet (education, wealth, VHW, religion); and soap and water (age, VHW). For maternal depressive symptoms: institutional delivery (education, wealth); breastfeeding initiation (none); late exclusive breastfeeding (age); child immunized (age, education, wealth); diverse diet (education, wealth); and soap and water (age). For gender norm attitudes, institutional delivery (education, wealth, religion); breastfeeding initiation (religion); late exclusive breastfeeding (age); child immunized (age, education, wealth, religion); diverse diet (education, wealth, religion); and soap and water (age). For mothering self-efficacy, institutional delivery (education, wealth, VHW, religion); breastfeeding initiation (religion); late exclusive breastfeeding (age); child immunized (age, education, wealth, VHW, religion); diverse diet (education, wealth, VHW, religion); and soap and water (age, VHW). For health status, institutional delivery (wealth, VHW, religion); breastfeeding initiation (religion); late exclusive breastfeeding (age); child immunized (age, wealth, VHW, religion); diverse diet (wealth, VHW, religion); and soap and water (age, VHW). For social support, institutional delivery (education, wealth, VHW); breastfeeding initiation (none); late exclusive breastfeeding (none); child immunized (education, wealth, VHW); diverse diet (education, wealth, VHW); and soap and water (VHW). For time stress, institutional delivery (education, wealth, VHW, religion); breastfeeding initiation (religion); late exclusive breastfeeding (age); child immunized (age, education, wealth, VHW, religion); diverse diet (education, wealth, VHW, religion); and soap and water (age, VHW). Abbreviations: VHW, village health worker; WASH, water, sanitation, and hygiene.

1 IYCF group only.

2 WASH group only.

## Discussion

We tested the hypothesis that mothers with stronger maternal capabilities during pregnancy will provide better care to their child between birth and 18 months of age. We found that 4 maternal capabilities (gender norm attitudes, depression, social support, and time stress) were significantly associated with at least 1 improved child-care practice. Decision-making autonomy was not associated with any care behavior, perhaps because decision-making autonomy scores were very high among nearly all women in SHINE. Perceived physical health was also not significantly associated with any care behavior. Though there was wide variability in perceived physical health, by objective indicators, SHINE women were in relatively good health [e.g., very few were undernourished, and 80% of those with HIV were receiving antiretroviral therapy (40)]. Future research could explore whether actual (rather than perceived) physical health is a determinant of child caregiving. In addition, contrary to its prominence in many health behavior models, mothering self-efficacy was only weakly associated with improved breastfeeding practices; stronger associations may be apparent between self-efficacy for more specific child-care behaviors and those behaviors, rather than “mothering” in general (51).

We observed strong predictive associations between egalitarian gender norm attitudes and positive child care across most of the practices we assessed. Previous research in LMICs on individual attitudes toward gender norms has largely focused on sexual and reproductive health outcomes [e.g., HIV/AIDS (52), family planning (53), and use of contraceptives (54)] and intimate partner violence (55). Our findings suggest that inequitable gender norms may not only be unjust for women and their health (56), but may also result in poorer child caregiving and child health. The pathways through which gender norm attitudes could impact child caregiving have not yet been clearly elucidated in the scientific literature.

Consistent with studies from South Asia (57), depression during pregnancy was strongly associated with 2 health care–seeking behaviors: depressed mothers were 40% less likely to deliver in a health institution, and their children were 33% less likely to be fully immunized. Antenatal screening for depression could identify these vulnerable women not only for referral to mental health services, but also for targeted visits by community-based health workers in order to facilitate institutional delivery and immunization receipt as a child survival intervention.

Social support was a significant predictor of institutional delivery and improved complementary feeding. Notably, women who reported being satisfied with the performance of their VHW had significantly greater perceived social support, in addition to higher mothering self-efficacy and less time stress. In many contexts throughout the world, community-based health workers (similar to the VHWs in this study) have been an effective platform for delivering complementary feeding behavior change (58); our finding suggests that the benefits of delivering services through community health workers may be mediated through improving social support.

Mothers with high levels of time stress were less likely to practice early EBF. Much of the breastfeeding literature has explored the competing demands of formal maternal employment on breastfeeding practices (59). However, since the large majority of SHINE mothers did not work outside the home, it is likely that their household work burden diverted them from breastfeeding, as has been reported in Bangladesh, where the high workload following the harvest season was associated with reduced breastfeeding time (60).

Household food security was significantly and substantially associated with all 7 maternal capabilities we assessed. This finding suggests that food insecurity may have negative effects on child well-being, not only through poorer child diets but also by reducing mothers’ capacity or motivation to make the best use of the limited resources that are available. Consistent with findings elsewhere, food insecurity was strongly associated with depressive symptoms (61–63). The causal direction, however, is not clear. In a longitudinal cohort study in Zambia, food insecurity, especially during the time of the year when households were typically food secure, was associated with subsequent depression. However, in a prospective cohort study of people living with HIV in Uganda, the initiation of antiretroviral therapy, which improved mental health, was associated with subsequent improved food security (64). It is possible that the relationship between food security and mental health is circular and mutually reinforcing.

While all of the 7 capabilities defined in our assessment tool were associated with food insecurity and most maternal education, other sociodemographic determinants varied between the capabilities. In addition, the 7 capabilities were only weakly associated with each other. These observations suggest that the 7 capabilities measured by our tool are conceptually and empirically distinct from each other, and likely reflect discrete aspects of a woman's capability to care for a child. Moreover, we observed substantial variability in maternal capabilities among SHINE women. Taken together, maternal capabilities, as assessed with these tools, may explain some of the heterogeneity in effectiveness of nutrition interventions within and between populations; these tools may also inform interventions to strengthen mothers’ capacity and motivation to provide optimal child care, which will likely be crucial in making further progress in reducing child undernutrition.

Strengths of this study include the rigorous cognitive interviewing within the study population in developing the tools, and the large sample size. Additionally, the longitudinal design allows for stronger inference about the directionality of the relationship between maternal capabilities and caregiving practices, compared to cross-sectional, observational design studies. Limitations include a lack of criterion validity against psychometric testing (except for depression). The cognitive interviewing completed in rural Zimbabwe did, however, provide face and content validity.

Our study provides tools that support efforts to harmonize assessments of maternal capabilities for the provision of optimal child care. In our study population, gender norm attitudes, mental health, social support, and time stress emerged as maternal factors associated with key child-care behaviors. Future research should test these tools in other contexts, elucidate the causal pathways between the capabilities and care behaviors, and, most importantly, identify and evaluate interventions to strengthen women's well-being and their capability to raise healthy children.

## Supplementary Material

nxaa255_Supplemental_File

## Notes

The Sanitation Hygiene Infant Nutrition Efficacy (SHINE) trial is funded by the Bill & Melinda Gates Foundation (OPP1021542 to the Johns Hopkins Bloomberg School of Public Health and OPP1143707 to the Zvitambo Institute for Maternal and Child Health Research), the UK Department for International Development, the Wellcome Trust (093768/Z/10/Z and 108065/Z/15/Z), the Swiss Agency for Development and Cooperation (8106727), and UNICEF (PCA-2017-0002).

Author disclosures: The authors report no conflicts of interest.

Supplemental Figure 1 is available from the ‘‘Online Supporting Material’’ link in the online posting of the article and from the same link in the online table of contents at http://jn.nutrition.org.

Abbreviations used: EBF, exclusive breast feeding; EPDS, Edinburgh Postnatal Depression Scale; IYCF, infant and young child feeding; LMIC, low- and middle-income country; SHINE, Sanitation Hygiene Infant Nutrition Efficacy; VHW, village health worker; WASH, water, sanitation, and hygiene.
