# Supplementary material for: Maternal Capabilities Are Associated with Child Caregiving Behaviors Among Women in Rural Zimbabwe
Source: J Nutr. 2020 Nov 19;151(3):685–94. doi: 10.1093/jn/nxaa255 (PMC7948208; doi:10.1093/jn/nxaa255)
Supplement: nxaa255_Supplemental_File [file nxaa255_supplemental_file.docx]

**5280** Pregnant women enrolled into SHINE trial

**1** additional pregnancy from a woman with 2 pregnancies

**11** pregnant women enrolled twice in error

**5270** Pregnant women enrolled from 211 clusters

**92** Exited without baseline visit

**16** Lost to follow up before baseline visit

**108** Miscarriage or still birth without a baseline visit

**57** missing baseline visit but had some follow up postpartum

**333** did not provide maternal capability data at baseline

**4667** Women provided maternal capability data at baseline

**67** Additional fetuses due to twin or triplet deliveries

**244** miscarried or had a still birth delivery

**35** Lost to follow up during prenatal period with unknown pregnancy outcome

**4455** Live births to women in 209 clusters

**1** Infant exited

**2** Maternal deaths

**176** Neonatal deaths

**4276** Live births eligible for 1-month postpartum visit

17 infants died between 1 and 3 months postpartum

**4259** Infants eligible for 3-month postpartum visit

46 Infants died between 3 and 12 months postpartum

3 Infants exited between 3 and 12 months postpartum

**4210** Infants eligible for 12-month postpartum visit
